# Difficulties in the use of medications by elderly people followed up in a cohort study in Southern Brazil

**DOI:** 10.1590/1980-549720230020

**Published:** 2023-03-10

**Authors:** Marília Cruz Guttier, Marysabel Pinto Telis Silveira, Noemia Urruth Leão Tavares, Matheus Carrett Krause, Renata Moraes Bielemann, Maria Cristina Gonzalez, Elaine Tomasi, Flavio Fernando Demarco, Andréa Dâmaso Bertoldi

**Affiliations:** Universidade Federal de Pelotas, Programa de Pós-Graduação em Epidemiologia –Pelotas (RS), Brasil.; Universidade Federal de Pelotas, Instituto de Biologia, Departamento de Fisiologia e Farmacologia, Programa de Pós-Graduação Multicêntrico em Ciências Fisiológicas – Pelotas (RS), Brasil.; Universidade de Brasília, Faculdade de Farmácia – Brasília (DF), Brasil.; Universidade Federal de Pelotas, Faculdade de Medicina – Pelotas (RS), Brasil.; Universidade Federal de Pelotas, Programa de Pós-Graduação em Nutrição e Alimentos – Pelotas (RS), Brasil.; Universidade Católica de Pelotas, Programa de Pós-Graduação em Saúde e Comportamento – Pelotas (RS), Brasil.

**Keywords:** Old age assistance, Elderly, Cohort studies, Drug utilization, Assistência a idosos, Idoso, Estudos de coortes, Uso de medicamentos

## Abstract

**Objective:**

This study aimed to assess the need for help by elderly people to take their medications, the difficulties related to this activity, the frequency of forgotten doses, and factors associated.

**Methods:**

Cross-sectional study conducted with a cohort of elderly people (60 years and over — “COMO VAI?” [How do you do?] study), where the need for help to properly take medication and the difficulties faced in using them were evaluated. The Poisson regression model was used to estimate the crude and adjusted prevalence ratios (PR) of the outcomes and respective 95% confidence intervals according to the characteristics of the sample.

**Results:**

In total, 1,161 elderly people were followed up. The prevalence of participants who reported requiring help with medication was 15.5% (95%CI 13.5–17.8), and the oldest subjects, with lower educational levels, in worse economic situations, on four or more medications and in bad self-rated health were the ones who needed help the most. Continuous use of medication was reported by 83.0% (95%CI 80.7–85.1) of the sample and most participants (74.9%; 95%CI 72.0–77.5) never forgot to take their medications.

**Conclusion:**

The need for help to use medications was shown to be influenced by social and economic determinants. Studies assessing the difficulties in medication use by the elderly are important to support policies and practices to improve adherence to treatment and the rational use of medications.

## RESUMO

**Objetivo:**

Este estudo visou avaliar a necessidade de ajuda dos idosos para tomar seus medicamentos, bem como as dificuldades relacionadas com a sua utilização, e a frequência de esquecimento de doses. Ainda, avaliar fatores associados à necessidade de ajuda dos idosos com os medicamentos.

**Métodos:**

Corte transversal em uma coorte de idosos (60 anos ou mais — estudo “COMO VAI?”), em que foi avaliada a necessidade de ajuda para tomar medicamentos de forma adequada e as dificuldades apresentadas na sua utilização. Utilizou-se regressão de Poisson para estimar as razões de prevalência (RP) brutas e ajustadas dos desfechos e seus intervalos de confiança de 95% (IC95%) de acordo com as características da amostra.

**Resultados:**

Participaram 1.161 idosos. A prevalência de idosos que relataram necessidade de ajuda com os medicamentos foi de 15,5% (IC95% 13,5–17,8), sendo que os mais idosos, com menor escolaridade e em pior situação econômica, em uso de quatro medicamentos ou mais e com pior autoavaliação de saúde foram os que mais necessitaram de ajuda. O uso contínuo de medicamentos foi referido por 83,0% (IC95% 80,7–85,1) e a maioria (74,9%; IC95% 72,0–77,5) nunca se esqueceu de tomar seus medicamentos.

**Conclusão:**

Observou-se a influência de determinantes sociais e econômicos e de saúde sobre a necessidade de ajuda para a utilização dos medicamentos. Estudos que estimem as dificuldades no uso de medicamentos por idosos são importantes para subsidiar políticas e práticas norteadoras de ações para melhorar a adesão e o uso racional de medicamentos.

## INTRODUCTION

Aging with quality of life is an important challenge when it comes to the care for the elderly. Policies have been developed seeking to promote health and aging with autonomy in the elderly population^1^, as well as to help caregivers, as there is an increase in the incidence of chronic diseases and, consequently, the need for medication to treat them^1–3^.

In Brazil, the prevalence of use of at least one continuous-use medication among the elderly ranges from 80 to 93%^4–6^. In Italy, this prevalence was similar (88%)^7^. Elderly people make use of multiple medications and are exposed to complex therapeutic regimes^6–11^, which can be unfavorable to treatment effectiveness.

Considering that the barriers to accessing health services and medication have been overcome and that the elderly have their drug treatment in hand, there are still other difficulties faced by them. Decline in cognitive status^12^, need for greater attention^13^, loss of visual acuity^14^ and loss of ability to handle medication packages^15^, as well as difficulties related to memory and time organization and management, can also be complicating factors for the correct use of medication^16^. In a cross-sectional study carried out in the city of Marília (SP), 59.8% of the elderly reported difficulties related to the use of medications, with forgetfulness being cited by a quarter of them^16^. A study carried out in Sweden pointed out that the majority (66.3%) of the elderly population had some limitation in the ability to manage their treatments^17^. Another difficulty cited by the elderly was the lack of belief in their efficacy^18^.

The main result of these difficulties is the lack of adherence to treatment, but they also contribute to errors in medication administration^16,17^, leading to unsatisfactory clinical results, adverse reactions and drug interactions^9,19^. Bearing in mind that the literature deals with these difficulties within adherence assessment scores^18,20,21^ or in studies assessing the instrumental activities of daily living (IADL) of the elderly^13,22–24^, this study aimed to assess the need for help by the elderly to take medications, as well as the difficulties faced when using them, and the frequency doses skipped or forgotten, after having overcome barriers to accessing health services and acquiring medication. Furthermore, the purpose was to evaluate factors associated with the need for help when taking medication at the correct dose and time.

## METHODS

Cross-sectional study with a cohort of elderly people, conducted in the urban area of the city of Pelotas, state of Rio Grande do Sul, Brazil (approximately 340,000 inhabitants in 2016). According to the Brazilian Institute of Geography and Statistics (IBGE)^25^, in 2010, 93% of the population of Pelotas lived in urban areas and approximately 50,000 were aged 60 years or older.

The sample recruitment and the first visit of the study called “COMO VAI?” (“How do you do?”) took place from January to August 2014. In total, 1,451 non-institutionalized elderly aged 60 years or older were included. The sampling process was carried out in two stages. Initially, clusters were selected using data from the 2010 Census^25^, with census groups being selected by lot. In the second stage, listed and systematically drawn households were selected—31 per sector—to enable the identification of at least 12 elderly people in each of them.

The second follow-up took place between November 2016 and April 2017, by telephone interviews; household visits were made in cases where telephone contact was not possible. Calls were made on different days and times, and participants not contacted by telephone had at least four visit attempts at the addresses made available to the study. The understanding of the questions was tested in a pilot study applied in face-to-face and telephone interviews.

Demographic, socioeconomic, and behavioral characteristics were the independent variables selected based on studies assessing adherence to treatment^18,20,21^ and IADL^13,22–24^. The following characteristics were collected in the first interview, to assist in the description of the sample: biological sex (male, female); age (60–69, 70–79, ≥80 years); skin color (self-reported, using the following categories: white, black, brown, yellow and indigenous, with the elderly self-declared as brown, yellow and indigenous grouped under the “mixed” category, due to the low frequency); education, defined as the highest level of education achieved in years of study (later categorized as none, <8 and ≥8 years); marital status (married/with a partner, single/divorced/widowed—considered “no partner”); economic situation (A/B — richer; and C, D/E — poorer), according to the criteria of the Brazilian Association of Companies and Research^26^.

Behavioral and health variables were also considered, given their importance in the evaluation of health care for the elderly. Characteristics such as current smoking (yes, no) were evaluated, considering daily cigarette consumption for more than one month; and alcohol consumption (yes, no), considering consumption of at least one dose of alcoholic beverage in the last 30 days. In addition, the concept of “polypharmacy” was evaluated, that is, simultaneous use of four or more medications^27^. Health perception was measured in 2016 by the question “How do you rate your health?”, with the following response options: very good, good, regular, poor and very poor, later recategorized as very good/good, fair, bad/very bad.

Outcomes were obtained at the second follow-up with the following filter question: “Do you need help taking your meds at the right dose and time?” (yes/no), which indicated the need for help with medication.

Among those who needed help with their treatments, the three outcomes related to difficulties in taking medications were evaluated using the following questions: “Thinking about your medication, could you tell me if the following actions are ‘very difficult’, ‘a little difficult’ or if ‘not difficult’?

- removing the medicine from package;
- reading the medicine package, to assess difficulties with handling, and understanding the package;
- taking too many medications at the same time, or difficulty with the amount of medications in use.

Continuous medication was also evaluated using the question “Do you take any continuous use medicine regularly, with no date to stop?” (Yes/No). For those who were on continuous medication, the following question was asked: a) “Do you sometimes forget to take your medicine?” (Yes/No); b) “How often do you have trouble remembering to take all your medications?”, with five response options: never/rarely, from time to time, sometimes, usually, always. Then, the responses were grouped into three categories (never/rarely, occasionally/sometimes, usually/always). These categories have been renamed to never, occasionally, and usually, respectively.

Only elderly people who met the outcome and were followed up at both moments were included in the analyses. The analytical sample maintained the characteristics of the original cohort, with the exception of age, since there was a significant decrease in the proportion of elderly aged 80 years or older (p=0.044) (Supplementary Table). Analyses were performed using the Stata statistical package, version 16.0 (Stata Corporation, College Station, USA). First, the sample was described (followed up in 2016 and 2017). Afterwards, the prevalence and 95% confidence intervals (95%CI) of the main outcome were obtained according to the characteristics of the sample. Poisson regression with forward selection was used to estimate the crude and adjusted prevalence ratios (PR), and the adjusted model included the variables that presented p<0.20 in the crude analysis to control for possible confounding factors. The respective 95%CI of each predictor's PR were estimated.

Descriptive analyses of the frequencies of outcomes were performed. Proportions were compared using the Pearson's χ2 test. Linear trend was assessed for significant associations between outcomes and exposure to more than two categories. The level of statistical significance was set at p<0.05.

The study was approved by the Research Ethics Committee of the Medical School of Universidade Federal de Pelotas—CAAE: 54141716.0.0000.5317. The participants or caregivers signed an informed consent form, guaranteeing data confidentiality. In 2016 and 2017, for the elderly interviewed by telephone, consent was provided verbally with acceptance to answer the questionnaire.

## RESULTS

The initial sample, in 2014, consisted of 1,451 elderly people. Of these, in 2016, 1,306 participants were located (145 obits identified). The follow-up rate was 90%, with the 1,161 elderly people who were alive being followed up. Most interviews (74.4%) took place over the phone.

Table 1 shows the analysis of the outcome “Need for help to take medication at the right dose and time”, according to demographic and socioeconomic characteristics in 2016. Most participants were females (63.7%) aged between 60 and 69 years (56.0%), white (83.6%), with less than 8 years of schooling (54.2%), married or with a partner (55.9%), and in level C economic status (57.6%). Altogether, 15.5% of the elderly (95%CI 13.5–17.8) reported needing help with medication use. There was no significant difference in the prevalence of help needed according to biological sex and skin color. Age, educational level and economic situation were important predictors for this outcome. The prevalence of elderly aged 80 years or older who reported needing help was 2.3 times higher (95%CI 1.6–3.5) than among subjects aged between 60 and 69 years, and 3.0 times higher (95%CI 1.6–5.4) among participants with no schooling, compared to those with 8 years or more of schooling. The prevalence of elderly people who reports needing help with their medications in economic strata D/E was 70% higher (PR=1.7; 95%CI 1.0–2.8) than among those in economic strata A/B. Marital status, after adjustment, lost statistical significance (Table 1).

**Table 1:** Sample description, with prevalence, crude and adjusted prevalence ratios of help needed to take medication at the right dose and time and respective 95% confidence intervals according to demographic and socioeconomic characteristics. Pelotas (RS), 2016.

	Sample n	Prevalence % (95%CI)	Crude PR 95%CI	Adjusted PR^*^ 95%CI
Sex			0.672	
Male	421 (36.3)	14.9 (11.7–18.7)	1.0	
Female	740 (63.7)	15.9 (13.4–18.7)	1.1 (0.8–1.5)	
Age (years)			<0.001	<0.001
60-69	648 (56.0)	10.1 (8.0–12.7)	1.0	1.0
70-79	363 (31.3)	17.4 (13.8–21.7)	1.7 (1.2–2.4)	1.3 (0.9–1.9)
80 and older	147 (12.7)	34.5 (27.1–42.7)	3.4 (2.3–4.9)	2.3 (1.6–3.5)
Skin color			0.285	
White	969 (83.6)	14.7 (12.5–17.1)	1.0	
Black	132 (11.4)	18.7 (12.9–26.5)	1.3 (0.8–2.0)	
Mixed	58 (5.0)	21.8 (12.8–34.6)	1.5 (0.8–2.7)	
Education (Years of study)			<0.001	0.002
None	147 (12.8)	30.7 (26.6–38.8)	5.5 (3.2–9.2)	3.0 (1.6–5.4)
Less than 8	625 (54.2)	17.8 (15.0–21.1)	3.2 (2.0–5.1)	2.3 (1.3–3.8)
8 and more	380 (33.0)	5.6 (3.7–8.5)	1.0	1.0
Marital Status			0.021	0.846
Partner	648 (55.9)	13.1 (10.7–15.9)	1.0	1.0
No partner	511 (44.1)	18.5 (15.4–22.2)	1.4 (1.1–1.9)	1.0 (0.7–1.3)
Economic status^†^			<0.001	0.029
A/B	311 (28.2)	10.5 (7.5–14.4)	1.0	1.0
C	634 (57.6)	14.0 (11.5–17.0)	1.3 (0.9–2.0)	1.0 (0.7–1.6)
D/E	156 (14.2)	30.9 (24.0–38.7)	3.0 (1.9–4.6)	1.7 (1.0–2.8)
Total		15.5 (13.5–17.8)		

* Analysis adjusted for age, education, marital status, economic status, alcohol consumption in the last 30 days, polypharmacy, and self-rated health;

† A/B — richest, C, D/E — poorest, according to the Brazilian Association of Companies and Research26; PR: prevalence ratio; 95%CI: 95% confidence interval.

Table 2 addresses the same outcome according to behavioral and health characteristics of participants. Most did not smoke (88.4%) or drink (76.5%), were under the concept of polypharmacy (53.7%), and perceived their health as very good or good (56.5%). Polypharmacy and self-rated health were important predictors for this outcome. The prevalence of elderly people who needed help was 1.6 times higher (95%CI 1.1–2.3) among those on four or more medications, compared to those on less than four medications. The worse the self-perception of health, the greater the need for help to take the medication, and among those who perceived their health as poor or very poor, the prevalence of help needed was 100% higher (PR=2.0; 95%CI 1.2–3.2) than among those who perceived their health as very good or good. Alcohol consumption in the last 30 days lost statistical significance after adjustment (Table 2).

**Table 2:** Description of the sample, prevalence, crude and adjusted prevalence ratios of help needed to take medication at the right dose and time with respective 95% confidence intervals according to behavioral characteristics. Pelotas (RS), 2016.

	Sample n	Prevalence % (95%CI)	Crude PR 95%CI	Adjusted PR^*^ 95%CI
Smoking currently		0.450	
No	1.024 (88.4)	15.8 (13.6–18.2)	1.2 (0.7–2.0)	
Yes	135 (11.6)	13.1 (8.3–20.0)	1.0	
Alcohol intake in the last 30 days			0.002	0.372
No	886 (76.5)	17.3 (14.9–20.0)	1.9 (1.2–2.8)	1.2 (0.8–2.0)
Yes	272 (23.5)	9.3 (6.4–13.4)	1.0	1.0
Polypharmacy			0.002	0.006
No	483 (46.3)	11.6 (9.0–14.8)	1.0	1.0
Yes	561 (53.7)	20.9 (17.7–24.5)	1.8 (1.3–2.5)	1.6 (1.1–2.3)
Self-rated health status			<0.001	0.012
Very good/good	654 (56.5)	9.9 (7.8–12.5)	1.0	1.0
Regular	414 (35.8)	20.4 (16.7–24.6)	2.1 (1.5–2.8)	1.5 (1.1–2.1)
Bad/very bad	89 (7.7)	35.4 (25.7–46.5)	3.6 (2.3–5.6)	2.0 (1.2–3.2)
Total		15.5 (13.5–17.8)		

* Analysis adjusted for age, education, marital status, economic situation, alcohol consumption in the last 30 days, polypharmacy, and self-rated health; PR: prevalence ratio; 95%CI: 95% confidence interval.

**Figure 1.**
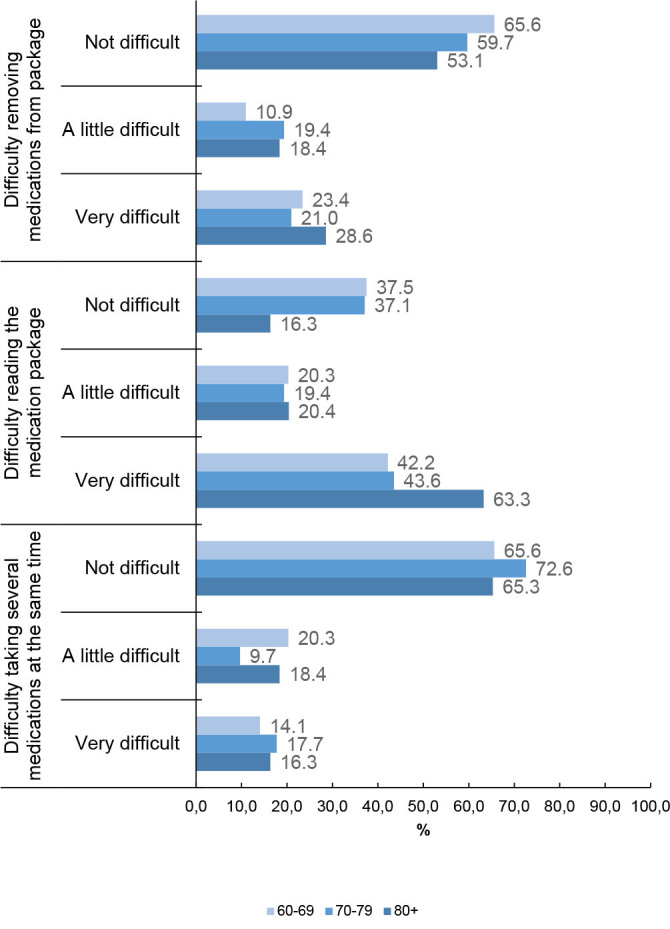
Level of difficulties faced by the elderly who reported needing help to use medication according to age (n=176). Pelotas (RS), 2016. Pearson's χ^2^ test to compare the proportions of each outcome with age.

Figure 1 shows the difficulties cited by the 176 elderly people who reported needing help to use medication, stratified by age. No significant difference was observed in the difficulty of removing medications from the package between age groups (p=0.55), to read package instructions (p=0.09) or to take many medications at the same time (p=0.55). For all ages, most participants do not find it difficult to unpack medications and take many at the same time. However, the most prevalent answer for reading the package was “very difficult” at all ages (Figure 1).

**Figure 2.**
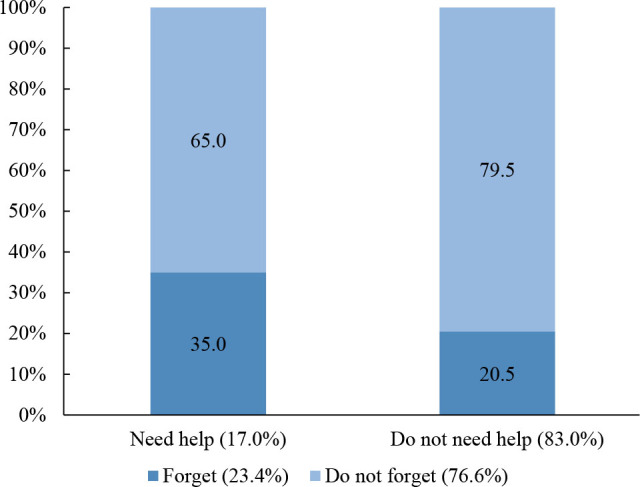
Proportion of elderly people on continuous medication who reported needing help to take their medications, according to the report of eventually forgetting doses (yes/no) (n=962) Pelotas (RS), 2016 (p<0.001). Pearson's χ^2^ test.

In assessing the use of continuous medication, 962 (83.0%; 95%CI 80.7–85.1) participants used them, among which 23.4% (95%CI 20.8–26, 1) reported occasionally forgetting to take doses. Figure 2 shows the proportion of elderly people on continuous medication who reported needing help to take them as forgetting is concerned. Among these users, 17.0% (95%CI 14.7–19.5) reported needing help and 83.0% (95%CI 80.5–85.3) reported not needing help. The proportion of forgetting doses among participants who needed help (35.0%) was significantly higher than among those who did not need help (20.5%; p<0.001) (Figure 2).

**Figure 3.**
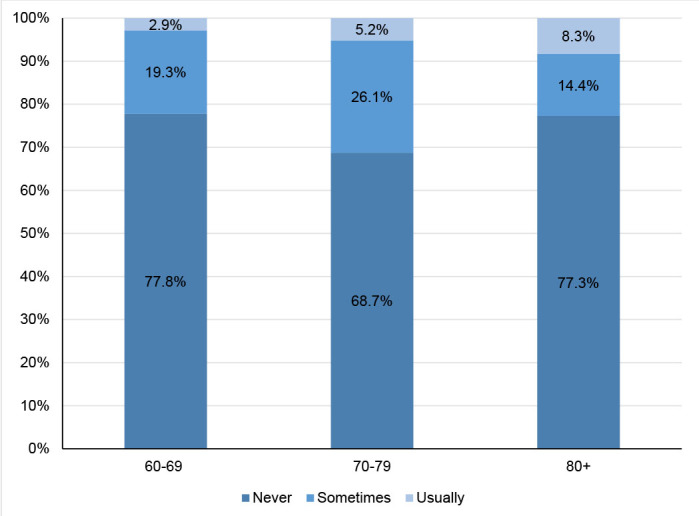
Frequency of forgetting medications according to age groups (n=959) Pelotas (RS), 2016 (p=0.002). Pearson's χ^2^ test to compare outcome proportions with age.

Figure 3 shows the frequency of forgetting doses according to age, for those on continuous medication. Among 956 users, most of them (74.9%; 95%CI 72.0–77.5) never forgot to take any doses. For the 60- 69 age group, 19.3% (95%CI 16.2–23.0) occasionally forget and 2.9% (95%CI 1.7–4.8), usually forget. In the age group 70-79 years old, 26.1% (95%CI 21.4–31.3) occasionally forget and 5.2% (95%CI 1.7–4.7), usually forget. Among the aged 80 years or older, 14.4% (95%CI 9.4–21.5) eventually forget and 8.3% (95%CI 4.7–14.4) usually forget (p=0.002) (Figure 3).

## DISCUSSION

This study shows that 15.5% of the elderly needed help to use their medication in the right dose and at the right time, and the greater the age, the lower the level of education and the worse the economic situation, the greater the proportion of elderly people who reported the need for help. Although there are methodological differences in studies that evaluate outcomes regarding the need for help and difficulty in using medications, in a population-based study carried out with elderly people aged 60 years or older in the city of São Paulo (SP), 8.5% of them had difficulty taking their medication and 89.3% received some sort of help in this task^28^.

The need for help with medications is a delicate issue, as when misused, they predispose the elderly population to the risks of polypharmacy and the possibility of developing more intense adverse or therapeutic effects, in addition to the likely increase in cost, both individually and for the health system^4^.

In addition, the need for help with medication can result in the need to expand the care network for the elderly and, in most cases, this network starts with family members, who leave aside their profession, leisure activities, and self-care to meet the needs of the elderly, often for prolonged periods, often until their deaths, which can lead to damage to the quality of life of the caregiver and the family^29^.

Another study, carried out in basic health units in the city of São Paulo (SP), used the Lawton Scale to identify the degree of dependence for IADL, and one of the evaluated items was whether the individual was able to take their medication in correct doses and in at correct times. It was observed that 46.8% of the elderly cannot, 28.2% need partial help, and only a quarter can use their medication without any help^24^.

Several factors are associated with impairment of functional capacity, such as advanced age, female gender, low income and education^24^. Low educational level was also associated with the inability to take medication in a descriptive study carried out with 95 elderly people treated at a Family Health Strategy (FHS) unit in Goiânia (GO)^13^, showing that adverse social and economic conditions negatively influence issues related to health, such as the need for help to use medications at correct dose and time. In that study, 30.0% of the elderly needed reminders to take their medications at the right time and 13.0% were unable to take them by themselves^13^.

The need for help from the elderly to deal with their treatments due to difficulty in handling medication packages, reading the packaging or taking too many medications directly interferes with adherence to treatment. Adherence to treatment is a complex, multifactorial matter that is essential for therapeutic results. When the patient does not adhere to treatment, there may be changes of various types, such as reduced benefits, increased risks, or both, which contributes to increased treatment costs for the elderly and for health services^30^. In this sense, understanding the factors that prevent the patient from following the recommendations of health professionals is important.

The need for help to take the medication in this study can be explained, in part, by difficulties in activities of daily living identified in the first follow-up, which were also associated with older age, lower education, and presence of multimorbidities^22^; however, this information was not collected in the follow-up from 2016, not allowing for these analyses. Considering that this is a longitudinal follow-up and aging being a limitation for the use of medication, it is likely that there will be an increase in the difficulties faced while using medication in the upcoming follow-ups.

Regarding the difficulties with the therapeutic regimen presented by the elderly who reported needing help, the greater difficulty was related to removing the medication from the package and reading it among elderly people aged 80 years or older. These difficulties may be associated with the loss of fine motor skills and reduced visual acuity in this population, although this study has not found a significant difference.

There is evidence that physiological aging can lead to decline in some tasks^31–33^. A systematic review aimed at analyzing factors associated with the autonomy of the elderly showed that the oldest (over 80 years old) are 40% more likely to let other people decide for them, when compared to those aged 60-69 years. That is, with aging, the probability of loss of autonomy increases, as well as the perception of autonomy worsens^34^.

Also, visual acuity can decrease with age and this can affect the ability of the elderly to read information on the medicine package, leading to errors or confusions, especially with those whose names are similar. A study carried out with 96 elderly people aged 65 and older from a community in the countryside of São Paulo showed a significant increase in the prevalence of low vision, compromising activities of daily living^35^.

Other important points refer to continuous medications, polypharmacy, and the self-perception of health. The elderly population lives with chronic health problems, being a great consumer of health services and medicines^36^, especially those for continuous use. This study showed that most elderly people aged 60 and older use this type of medication and that polypharmacy and poorer health perception were also associated with a greater need for help with medication. The high prevalence of polypharmacy among the elderly population points to the importance of identifying the needs of this population in order to make rational use of treatment^37,38^.

However, the results of this study showed that, of those on continuous medication, about a quarter eventually forget to take their medication, although most of them reported never forgetting (74.9%; 95%CI 72.0– 77.5). These results were lower than those reported by Bezerra et al.^39^ and Rocha et al.^5^, and higher than those reported by Marin et al.^16^ Forgetting is a serious problem, as it can directly impact adherence to treatment and, consequently, the effectiveness of medications, leading to unsatisfactory control of multimorbidities^40^. It is estimated that, in high-income countries, adherence to long-term therapies accounts to only 50% on average. In middle-income countries, the rates are even lower, which seriously compromises the efficacy of treatments and has important implications in quality of life, the economy, and public health^1^.

One of the limitations are the impossibility of collecting all behavioral and health characteristics in the same follow-up in which the outcome was collected, which may have underestimated or overestimated the relation of these variables with the outcome, even though the interval between follow-ups was of only two years. Not having evaluated the functional limitations of the elderly can also be a limitation, as these characteristics can directly influence the outcomes. However, the study has strengths: a population-based longitudinal study sample was used, with frequent follow-ups; however, hospitalized or institutionalized elderly were not included in the study. Even working with the elderly and the study not being initially planned to have a cohort design, the follow-up rate was high.

Social and economic determinants were found to influence on the elderly's need for help to use their medications, and a high prevalence of elderly people on continuous treatment (with a quarter of these forgetting to take doses eventually, significantly higher among those who need help). Studies that estimate the difficulties faced with medications by the elderly are important to support health policies and practices aimed at minimizing issues and guiding actions to improve adherence to treatment and rational use of medication.
